# Integrated Metabolomics and Transcriptomics Analysis of Monolayer and Neurospheres from Established Glioblastoma Cell Lines

**DOI:** 10.3390/cancers13061327

**Published:** 2021-03-16

**Authors:** Joana Peixoto, Sudha Janaki-Raman, Lisa Schlicker, Werner Schmitz, Susanne Walz, Alina M. Winkelkotte, Christel Herold-Mende, Paula Soares, Almut Schulze, Jorge Lima

**Affiliations:** 1 Cancer Signalling and Metabolism Group, Instituto de Investigação e Inovação em Saúde (i3S), Universidade do Porto, 4200-135 Porto, Portugal; jpeixoto@ipatimup.pt (J.P.); psoares@ipatimup.pt (P.S.); 2 Cancer Signalling and Metabolism Group, Institute of Molecular Pathology and Immunology of the University of Porto (Ipatimup), 4200-465 Porto, Portugal; 3 Faculty of Medical, University of Porto, 4200-319 Porto, Portugal; 4 Department of Biochemistry and Molecular Biology, Theodor-Boveri-Institute, Biocenter, Am Hubland, 97074 Würzburg, Germany; JanakiRS@mskcc.org (S.J.-R.); wschmitz@biozentrum.uni-wuerzburg.de (W.S.); susanne.walz@biozentrum.uni-wuerzburg.de (S.W.); almut.schulze@dkfz-heidelberg.de (A.S.); 5 Division of Tumor Metabolism and Microenvironment, German Cancer Research Center, Im Neuenheimer Feld 581, 69120 Heidelberg, Germany; l.schlicker@dkfz-heidelberg.de (L.S.); a.winkelkotte@dkfz-heidelberg.de (A.M.W.); 6 Faculty of Biosciences, University of Heidelberg, 69120 Heidelberg, Germany; 7 Division of Neurosurgical Research, Department of Neurosurgery, University of Heidelberg, 69117 Heidelberg, Germany; h.mende@med.uni-heidelberg.de

**Keywords:** glioblastoma, neurospheres, monolayer, metabolome, transcriptome, arginine metabolism

## Abstract

**Simple Summary:**

Glioblastomas are very aggressive tumours without efficient treatment, where cancer stem-like cells are thought to be responsible for relapse. This pilot study investigated the metabolic discrepancies between monolayer and neurosphere cultures of two glioblastoma cell lines using transcriptomics and metabolomics. We show that the two culture systems display substantial differences regarding their metabolome and transcriptome. Specifically, we found that metabolic reactions connected to arginine biosynthesis are crucial to support the different metabolic needs of neurospheres from the two cell lines. By identifying metabolic vulnerabilities in different glioblastoma subpopulations, new therapeutic strategies may be emerging that can be explored to treat this disease. Moreover, this data set may be of great value as a resource for the scientific community.

**Abstract:**

Altered metabolic processes contribute to carcinogenesis by modulating proliferation, survival and differentiation. Tumours are composed of different cell populations, with cancer stem-like cells being one of the most prominent examples. This specific pool of cells is thought to be responsible for cancer growth and recurrence and plays a particularly relevant role in glioblastoma (GBM), the most lethal form of primary brain tumours. Here, we have analysed the transcriptome and metabolome of an established GBM cell line (U87) and a patient-derived GBM stem-like cell line (NCH644) exposed to neurosphere or monolayer culture conditions. By integrating transcriptome and metabolome data, we identified key metabolic pathways and gene signatures that are associated with stem-like and differentiated states in GBM cells, and demonstrated that neurospheres and monolayer cells differ substantially in their metabolism and gene regulation. Furthermore, arginine biosynthesis was identified as the most significantly regulated pathway in neurospheres, although individual nodes of this pathway were distinctly regulated in the two cellular systems. Neurosphere conditions, as opposed to monolayer conditions, cause a transcriptomic and metabolic rewiring that may be crucial for the regulation of stem-like features, where arginine biosynthesis may be a key metabolic pathway. Additionally, TCGA data from GBM patients showed significant regulation of specific components of the arginine biosynthesis pathway, providing further evidence for the importance of this metabolic pathway in GBM.

## 1. Introduction

The cancer stem cell concept is based on the scientific discovery that tumours, like normal tissues, are composed of subpopulations of cells that can both self-renew and differentiate into several different cell populations. These cells can drive tumour initiation, maintenance and progression, as well as cancer recurrence and metastasis. Specifically in glioblastoma (GBM) models, both in vitro and in vivo studies have identified the existence of glioblastoma stem-like cells (GSCs) expressing stem cell-related markers, which are able to initiate and recapitulate tumour heterogeneity when injected orthotopically into mice [1,2]. In this context, GSCs are defined as tumour cells with unlimited self-renewal potential, multipotent differentiation capacity and high tumourigenic ability [1]. Moreover, GSCs have been defined as mediators of therapy resistance by circumventing radio- and chemotherapy and by driving invasion, angiogenesis and recurrence [3,4,5,6,7,8]. Besides, GSCs can be functionally distinguished from their differentiated tumour progeny at core levels ranging from transcription and epigenetics to metabolic regulation. The high degree of intratumoural heterogeneity, along with the infiltrative, migratory and plastic nature of GBM cells, as well as the high recurrence rate of this disease, represent the major contributors for the poor prognosis of GBM patients.

The available in vitro methods to isolate and/or enrich for GSCs involve using defined suspension cultures to grow and propagate GBM cells, thereby distinguishing them from the non-stem compartment [9,10]. This is known as the neurosphere (NS) formation assay, which depends on the self-renewal properties of GSCs to allow their survival in defined non-adherent conditions. Using primary human GBMs, it was reported that cells propagated in vitro under defined culture conditions could form NS enriched for cells exhibiting typical GSC characteristics: potential for indefinite self-renewal and capacity for terminal differentiation into glial and neuronal lineages [2]. Moreover, these cells displayed gene expression profiles and biological behaviour that closely mimic their parental primary tumours, in contrast with tumour cells grown under standard culture conditions. In fact, standard culture conditions were not able to enrich for GSCs, ultimately leading to the propagation of a population of cells that remotely resembled their parental tumours [2].

As a disease with a very high demand for building blocks for rapid cell proliferation and survival, cancer is characterised by profound metabolic remodelling that drives the accumulation of metabolic intermediates, which are then used as a source for biomass production. However, it has become evident that besides providing energetic and biomass supply, altered metabolic circuits also exert effects on shaping transformation and cell fate, ultimately controlling the establishment and maintenance of the tumourigenic state of a cancer cell. Moreover, many studies in the cancer metabolism field have so far not considered that most tumours contain metabolically distinct cell populations and that this might impose a challenge for metabolism-targeting treatments.

In this study, we have integrated metabolomics and transcriptomics data to identify key metabolic pathways in GBM cells cultured in NS conditions, as opposed to monolayer (ML) cultures. We unveiled that NS cultures, when compared with ML cultures, resulted in the downregulation of differentiation-related gene signatures, which supports the applicability of these culture conditions for GSCs studies. By making use of two different cellular systems, an established GBM cell line adapted to grow in ML conditions (U87) and patient-derived GBM stem-like cells adapted to grow under NS conditions (NCH644), we have provided evidence that NS cultures cause a profound metabolic rewiring in both cellular systems. Furthermore, we found that arginine biosynthesis was the most significantly regulated pathway in NS cultures in both cell systems. Nevertheless, individual nodes of this pathway showed distinct regulation in the two cell lines, revealing that different metabolic fates caused by the expression of specific key players of the same pathway can ultimately support distinct metabolic needs in different cancer cell lines. Importantly, specific enzymes of the arginine biosynthesis pathway were also found to be deregulated in publicly available datasets from human GBM, highlighting the potential importance of this metabolic process in GBM.

## 2. Results

### 2.1. Transcriptomic Profile of Monolayer and Neurospheres

**Figure 1:**
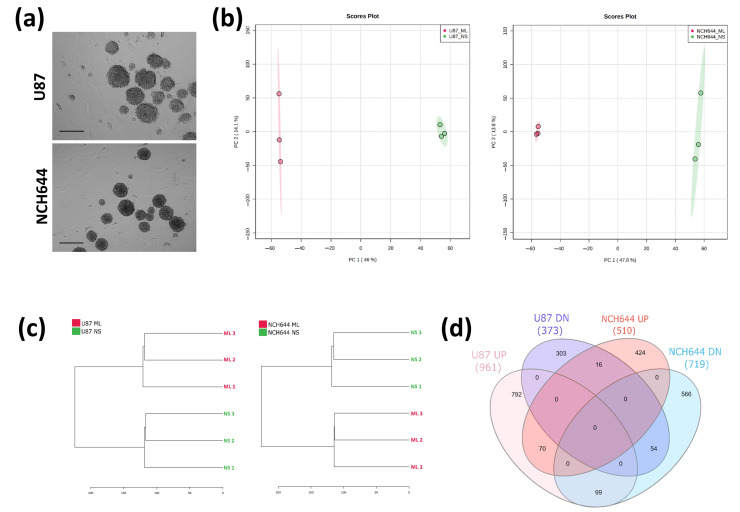

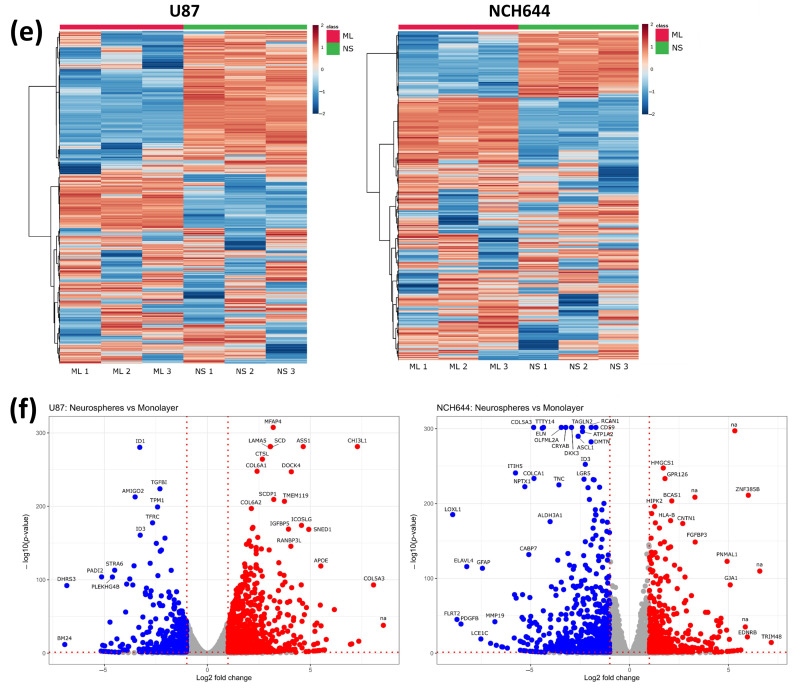
Transcriptomics analysis of monolayer (ML) and neurospheres (NS) from U87 and NCH644 cells. (**a**) Representative image of U87 and NCH644 NS cultured for four days, before cell harvesting for RNA and metabolite extraction. Scale bar = 150 µm; (**b**) Principal component analysis and (**c**) hierarchical clustering of all genes, showing a clear separation between ML and NS samples in both cell lines. (**d**) Venn diagram depicting the number of significantly upregulated (UP) and downregulated (DN) genes in NS vs. ML, as well as the number of commonly and differentially regulated genes between U87 and NCH644 cells (FDR < 0.05, log2FC > ±1). (**e**) Heatmaps and (**f**) volcano plots of upregulated (red) and downregulated (blue) genes in NS vs. ML from U87 and NCH644 cells (FDR < 0.05, log2FC > ±1); *n* = 3.

To identify changes in gene expression in GBM cells exposed to different culture conditions that promote either a stem-like or differentiated phenotype, U87 and NCH644 cells were exposed to neurosphere (NS) and monolayer (ML) culture conditions (Figure 1a) and total RNA was prepared. Transcriptomics analysis by RNA sequencing identified a total of 18,457 genes being expressed across all conditions. Principal component analysis (PCA) revealed a global transcriptomic rewiring between ML and NS in both cell lines (Figure 1b), as PC1 accounted for 46% of all variability in U87 cells and nearly 48% in NCH644 cells. The same is also apparent from the hierarchical clustering (Figure 1c), suggesting that cells exposed to the different culture conditions clearly diverge in their gene expression profile. In total, 1334 differentially expressed genes were identified in U87 cells and 1229 genes in NCH644 cells with a false discovery rate (FDR) < 0.05 and a log2FC > ±1 (Table S1a,b). The comparison between NS and ML in U87 and NCH644 presented 961 and 510 upregulated genes and 373 and 719 downregulated genes, respectively (Figure 1d). From these, 124 genes were commonly regulated by both U87 and NCH644 cells: 70 commonly upregulated genes and 54 commonly downregulated genes in NS over ML cells (Figure 1d). Moreover, 115 genes were differentially expressed in NS of the two cell lines: 16 genes were upregulated in NCH644 and downregulated in U87 cells, while 99 genes were upregulated in U87 and downregulated in NCH644 cells (Figure 1d). Corresponding heat maps and volcano plots are also shown (Figure 1e,f).

To gain further insight into the processes that are differentially regulated between ML and NS conditions, gene set enrichment analysis (GSEA) was performed using gene sets assigned to crucial biological processes, namely the C2 collection for curated gene sets, C5 for gene ontology gene sets and H for hallmark gene sets (www.gsea-msigdb.org, accessed on 27 September 2018). FDR values were considered to indicate significant enrichment when smaller than 0.25. 

**Figure 2:**
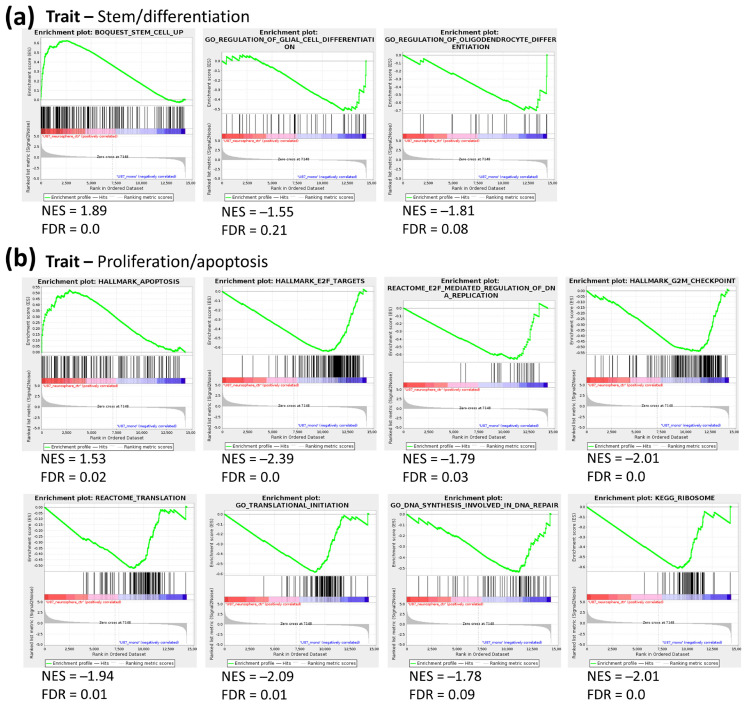

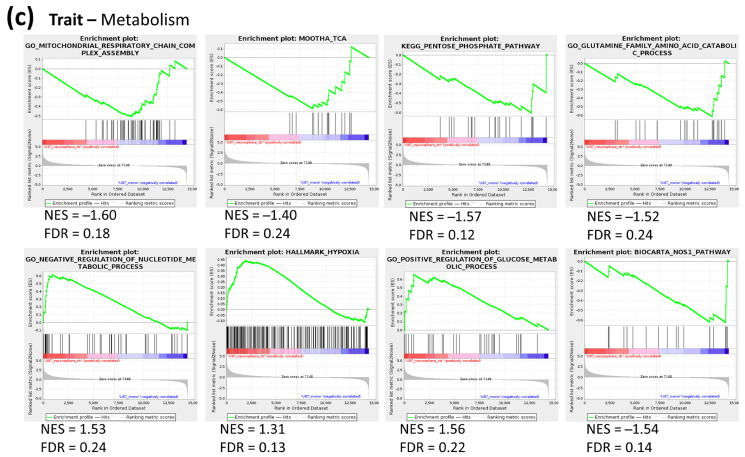
Gene set enrichment analysis (GSEA) of gene sets associated with stem/differentiation, proliferation/apoptosis and metabolism traits in NS vs. ML from U87 cells. (**a**) A stem cell-associated gene set is upregulated in NS, while two differentiation gene sets are enriched in ML (**b**) An apoptosis gene set is enriched, while several proliferation-associated gene sets are downregulated in NS. (**c**) Metabolism-associated gene sets, namely mitochondrial respiratory chain assembly, TCA cycle, PPP and glutamine metabolism are downregulated in NS, while a gene set representing negative regulation of nucleotide metabolism is upregulated. Hypoxia and glucose metabolism gene sets are upregulated, while a nitric oxide pathway gene set is downregulated in NS; *n* = 3, FDR < 0.25.

**Figure 3:**
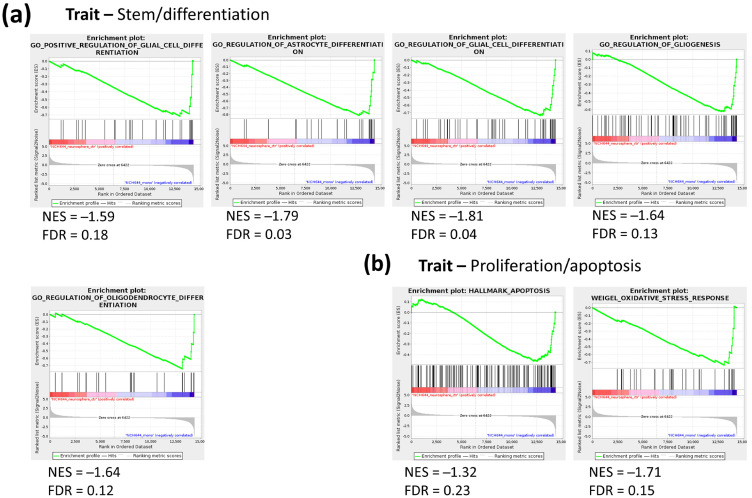
GSEA of gene sets associated with stem/differentiation and proliferation/apoptosis traits in NS vs. ML from NCH644 cells. (**a**) Several differentiation-associated gene sets are enriched in NS. (**b**) Gene sets associated with apoptosis and oxidative stress response are downregulated in NS; *n* = 3, FDR < 0.25.

GSEA revealed 292 C2 gene sets, 143 C5 gene sets and 21 H gene sets that show significantly higher expression in U87 NS compared to ML (Table S1c). On the other hand, 238 C2 gene sets, 157 C5 gene sets and 4 H gene sets showed higher expression in ML compared to NS (Table S1c). Surprisingly, in NCH644 cells, GSEA showed no gene sets that were significantly induced in NS compared to ML, while 50 C2 gene sets, 142 C5 gene sets and 8 H gene sets were found to be upregulated in ML over NS (Table S1d). From these, gene sets assigned to general traits such as metabolism, proliferation/apoptosis and stem/differentiation processes were selected, and the respective enrichment plots are displayed (Figure 2 and Figure 3). In U87 cells, a previously defined stem cell signature showed clear enrichment in the NS condition, while ML cells presented an enrichment of glial cell and oligodendrocyte differentiation signatures (Figure 2a). This strongly suggests that NS culture conditions indeed induce a stem-like phenotype, while ML culture conditions maintain cells under a more differentiated state. Moreover, U87 NS cultures showed enrichment of an apoptosis gene signature, while several signatures associated with cell cycle, translation and DNA replication/repair were downregulated (Figure 2b), suggesting that U87 cells in NS culture have lower proliferative capacity than their ML counterparts. Gene sets representing metabolic pathways, including mitochondrial respiratory chain assembly, tricarboxylic acid (TCA) cycle, pentose phosphate pathway (PPP) and glutamine metabolism were down in U87 NS (Figure 2c), while a gene set representing negative regulation of nucleotide metabolism was upregulated (Figure 2c), supporting metabolic rewiring driven by altered gene expression. In contrast, two gene sets associated with hypoxia and regulation of glucose metabolism were upregulated in U87 NS (Figure 2c), and both processes have already been associated with stem-like features in GBM [11,12,13]. Additionally, a gene set associated with the nitric oxide pathway was downregulated in U87 NS, pointing at a possible regulation of nitric oxide synthesis in these cells (Figure 2c). 

While no gene sets were enriched in NS cultures in NCH644 cells, several gene sets mapping to glial differentiation were upregulated in ML cultures (Figure 3a), again supporting that this culture condition enriches for cell differentiation. In contrast to U87, which displayed an enrichment of an apoptosis gene signature in NS, NCH644 showed a downregulation of the same gene signature in NS cultures, along with the downregulation of an oxidative stress gene set (Figure 3b). This suggests that NCH644 cells exposed to NS conditions may not be under the same growth restrictions as U87 cells. Altogether, the results indicate that NCH644 cells undergo a less substantial restructuring of their transcriptional activity when exposed to the different culture conditions, as fewer gene sets were found to be significantly regulated, compared to U87. Nevertheless, both cell lines showed differential expression of gene signatures associated with glial differentiation, thus constituting suitable models to study cellular processes under stem-like and differentiation conditions.

### 2.2. Metabolic Profile of Monolayer and Neurospheres

**Figure 4:**
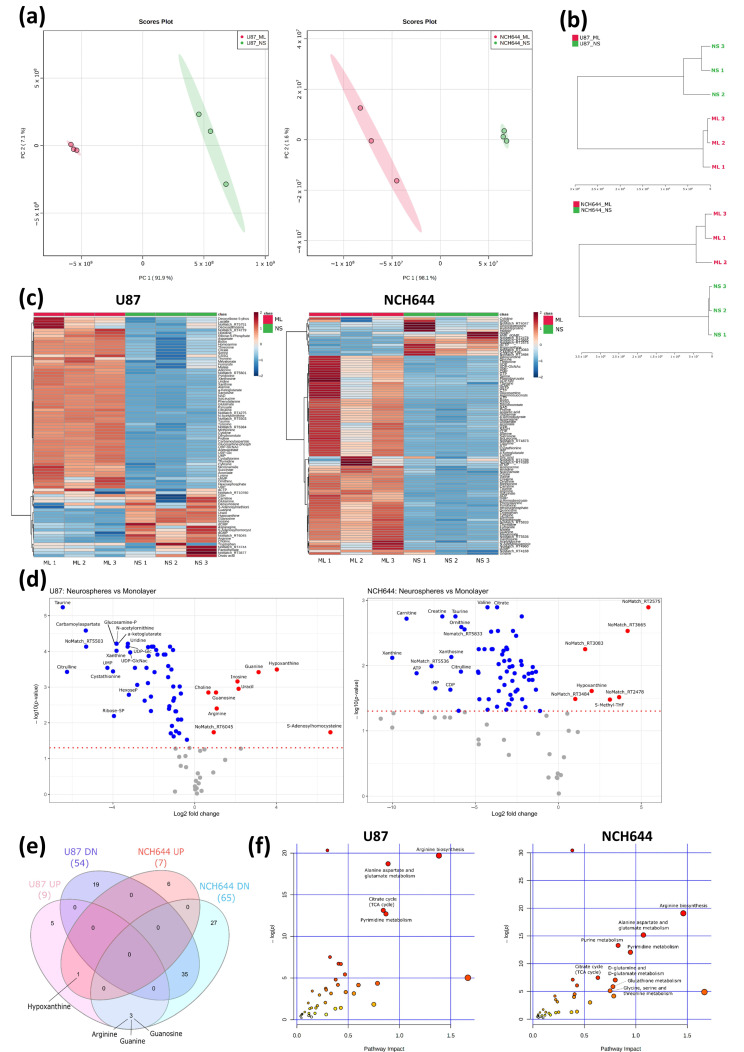
Metabolomics analysis of ML and NS from U87 and NCH644 cells. (**a**) Principal component analysis and (**b**) hierarchical clustering of all metabolites, showing a clear separation between ML and NS samples in both cell lines. (**c**) Heatmaps and (**d**) volcano plots of increased (red) and decreased (blue) metabolite levels in NS vs. ML from U87 and NCH644 cells (FDR < 0.05). (**e**) Venn diagram depicting the number of significantly increased (UP) and decreased (DN) metabolites in NS vs. ML, as well as commonly and differentially regulated metabolites between U87 and NCH644 cells (FDR < 0.05). (**f**) Metaboanalyst analysis of significantly regulated metabolites in NS vs. ML from U87 and NCH644 cells (FDR < 0.05, Pathway impact > 0.5); *n* = 3.

To analyse general metabolic differences between ML and NS conditions, water-soluble metabolites were extracted from cells exposed to these culture conditions and analysed by LC-MS. Using TraceFinder and an in-house metabolite library, we identified a total of 84 metabolites in U87 cells and 99 metabolites in NCH644 cells. PCA based on these data clearly separated ML from NS conditions in both cell lines, with PC1 accounting for nearly 92% of the variability in U87 cells and 98% of the variability in NCH644 cells (Figure 4a). The strong separation along PC1 based on metabolite data could indicate that the different culture conditions cause more pronounced changes in the metabolome compared to those observed in the transcriptome (Figure 1b). Similar conclusions are also supported by hierarchical clustering of the samples based on metabolite data (Figure 1c and Figure 4b).

Statistical analysis of the metabolome data set identified 63 metabolites significantly altered in ML vs. NS from U87 cells and 72 metabolites in ML vs. NS from NCH644 cells (Table S1e,f). Heat maps and volcano plots for these analyses are shown in Figure 4c,d. Interestingly, both cell lines presented only a small number of metabolites that were more abundant in NS compared to the ML condition (9 in U87 and 7 in NCH644, respectively). In contrast, a large number of metabolites showed significant downregulation in NS compared to ML (54 in U87 and 65 in NCH644) (Figure 4c–e). Out of the downregulated metabolites, 35 were commonly regulated in both cell lines, while only one metabolite—hypoxanthine—was more abundant in NS compared to ML in both cell lines (Figure 4e). These results indicate that NS conditions cause a metabolic rewiring towards reduced metabolite availability which points to a lower cellular metabolic activity. Additionally, three metabolites were regulated differentially in the two cell lines: U87 NS presented significantly higher levels of arginine, guanine and guanosine, in comparison with ML cells, while NCH644 NS had significantly lower levels of the same metabolites compared to ML (Figure 4e).

To identify specific metabolic pathways regulated by the different culture conditions, we performed a pathway analysis on significantly altered metabolites in MetaboAnalyst and selected those pathways that showed an FDR lower than 0.05 and a pathway impact above 0.5 (Table S1g,h). This analysis showed that four metabolic pathways were commonly regulated in both U87 and NCH644 ML vs. NS cultures. These were: “Arginine biosynthesis”, “Alanine, aspartate and glutamate metabolism”, “Citrate cycle (TCA cycle)” and “Pyrimidine metabolism” (Figure 4f). Additionally, the pathways “Purine metabolism”, “D-glutamine and D-glutamate metabolism”, “Glutathione metabolism” and “Glycine, serine and threonine metabolism” were uniquely regulated in NCH644 ML vs. NS cells (Figure 4f). This suggests that these commonly regulated pathways could be crucially involved in the induction and/or maintenance of the NS phenotype. On the other hand, uniquely regulated pathways could represent specific cell line features that may not reflect general NS traits.

### 2.3. Joint Metabolomics and Gene Expression Pathway Analysis of Monolayer and Neurospheres

**Figure 5:**
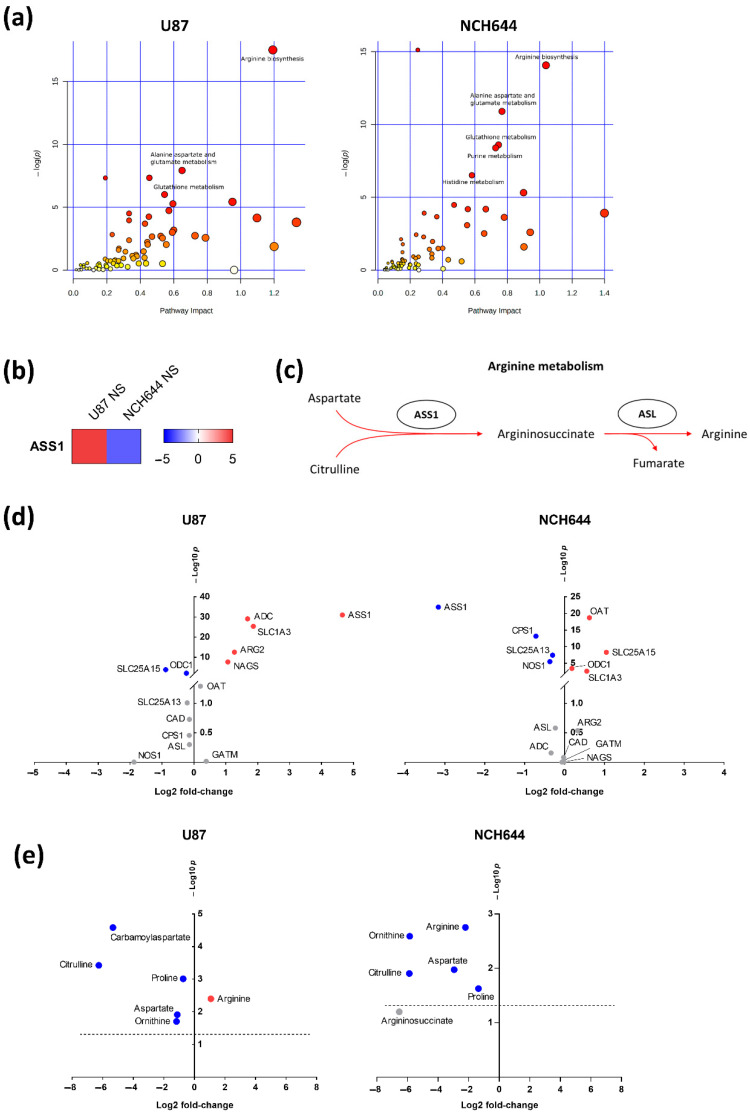
Joint pathway analysis of NS vs. ML reveals arginine biosynthesis as the most significantly regulated pathway. (**a**) Joint pathway analysis of significantly regulated metabolites (FDR < 0.05) and genes (FDR < 0.05, log2FC > ±1) in NS vs. ML from U87 and NCH644 cells. (**b**) *ASS1* is significantly upregulated in U87 NS and downregulated in NCH644 NS, compared to ML cells (FDR < 0.05). (**c**) Schematic view of the arginine biosynthesis process. (**d**) Significantly upregulated (red) and downregulated (blue) genes involved in arginine metabolism, in NS vs. ML from U87 and NCH644 cells (FDR < 0.05). (**e**) Metabolites involved in arginine metabolism whose levels are significantly increased (red) and decreased (blue), in NS vs. ML from U87 and NCH644 cells (FDR < 0.05); *n* = 3. ADC—arginine decarboxylase; ARG—arginase; ASL—argininosuccinate lyase; ASS1—argininosuccinate synthase; CAD—carbamoyl-phosphate synthetase 2, aspartate transcarbamylase and dihydroorotase; CPS1—carbamoyl-phosphate synthase 1; GATM—Glycine Amidinotransferase; NAGS—N-acetylglutamate synthase; NOS1—nitric oxide synthase 1; OAT—ornithine aminotransferase; ODC1—ornithine decarboxylase 1; SLC1A3—solute carrier family 1 member 3; SLC25A13—solute carrier family 25 member 13; SLC25A15—solute carrier family 25 member 15.

To define the most relevant metabolic pathways regulated in NS vs. ML cells, a joint pathway analysis was performed in MetaboAnalyst by combining metabolomics and gene expression data. Only significantly altered genes and metabolites were included in this analysis (FDR < 0.05). Again, metabolic pathways with an FDR lower than 0.05 and a pathway impact above 0.5 were selected for further consideration (Table S1i,j). Three common pathways were found to be regulated in both U87 and NCH644 ML vs. NS cultures: “Arginine biosynthesis”, “Alanine, aspartate and glutamate metabolism” and “Glutathione metabolism” (Figure 5a). Furthermore, NCH644 ML vs. NS cells presented additional uniquely regulated pathways: “Purine metabolism” and “Histidine metabolism”. Since arginine metabolism was the most significantly regulated pathway in both cell lines, we analysed the individual components within this metabolic process in more detail. By evaluating the expression of significantly regulated genes involved in arginine biosynthesis, we found that U87 NS present a strong upregulation of argininosuccinate synthase (ASS1) (log2FC = 4.65), while NCH644 NS displayed a strong downregulation of this gene (log2FC = −3.16) (Figure 5b). ASS1 catalyses the rate-limiting step for arginine biosynthesis in the urea cycle by combining two amino acids, citrulline and aspartate, to form argininosuccinate (Figure 5c). We therefore evaluated the expression of other enzymes and metabolites involved in urea cycle metabolism in both cell lines. We observed that multiple urea cycle components were significantly altered between NS and ML cultures in the two cell lines (Figure 5d,e). In particular, U87 NS showed an upregulation of the genes coding for arginine decarboxylase (ADC) (log2FC = 1.68), solute carrier family 1 member 3 (SLC1A3) (log2FC = 1.86), arginase 2 (ARG2) (log2FC = 1.27) and N-acetylglutamate synthase (NAGS) (log2FC = 1.07), while a downregulation of ornithine decarboxylase 1 (ODC1) (log2FC = −0.24) and solute carrier family 25 member 15 (SLC25A15) (log2FC = −0.88) was observed (Figure 5d). Concomitantly, U87 NS presented significantly higher levels of arginine (log2FC = 1.08) and lower levels of carbamoylaspartate (log2FC = −5.33), proline (log2FC = −0.73), ornithine (log2FC = −1.17), citrulline (log2FC = −6.25) and aspartate (log2FC = −1.11) (Figure 5e). In contrast, NCH644 NS showed upregulation of the genes coding for ornithine aminotransferase (OAT) (log2FC = 0.62), ODC1 (log2FC = 0.19), SLC25A15 (log2FC = 1.05) and SLC1A3 (log2FC = 0.56), with downregulation of carbamoyl-phosphate synthase 1 (CPS1) (log2FC = −0.72), nitric oxide synthase 1 (NOS1) (log2FC = −0.37) and solute carrier family 25 member 13 (SLC25A13) (log2FC = −0.30) (Figure 5d). Additionally, NCH644 NS presented significantly lower levels of arginine (log2FC = −2.21), ornithine (log2FC = −5.86), aspartate (log2FC = −2.95), citrulline (log2FC = −5.88) and proline (log2FC = −1.36) (Figure 5e).

### 2.4. Differential Expression of Arginine Biosynthesis Genes in Human GBM and GBM Cell Lines

**Figure 6:**
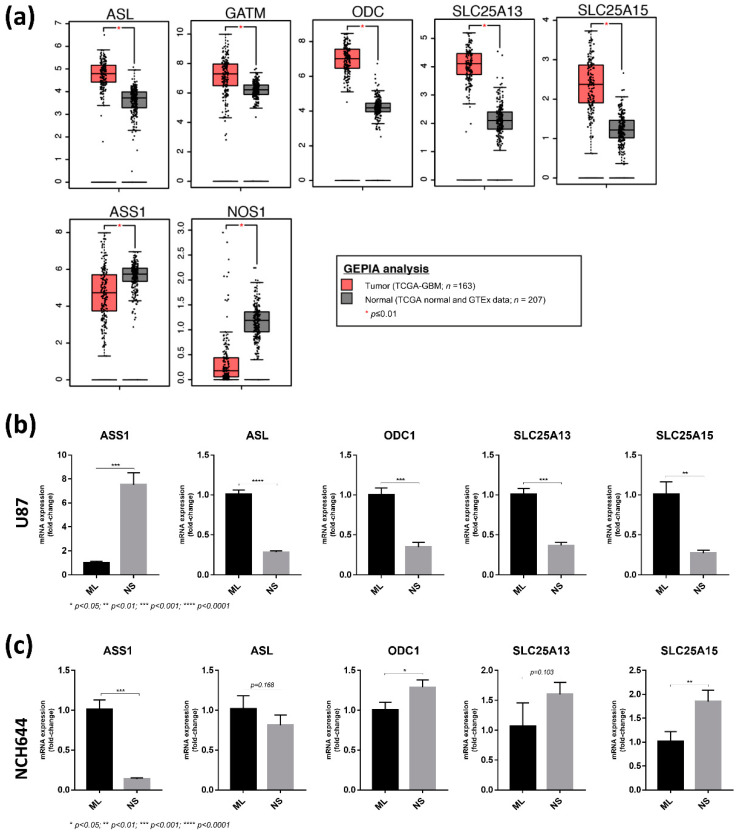
Expression analysis of arginine biosynthesis genes in NCH644 NS compared to ML cells may resemble human GBM tumours compared to normal tissue. (**a**) Expression data from human GBM tumours (TCGA-GBM; *n* = 163) and corresponding normal tissue (TCGA normal and GTEx data; *n* = 207) were accessed via the GEPIA tool (http://gepia.cancer-pku.cn/index.html, accessed on the 3 December 2020). Data were selected based on a log2-fold change cut-off of 1 and a *p*-value cut-off of 0.01. Only significantly altered genes are shown. (**b**) qPCR analysis of arginine biosynthesis genes in U87 ML and NS cultures. (**c**) qPCR analysis of arginine biosynthesis genes in NCH644 ML and NS cultures.

As we identified genes and metabolites of the arginine biosynthesis pathway to be differentially regulated between ML and NS cultures in the two cell lines, we next investigated whether alterations in expression of these genes can also be found in the human disease. For this, we accessed data from The Cancer Genome Atlas (TCGA) via the GEPIA analysis tool [14]. Out of the 14 genes analysed, we found that 7 showed significant differences in expression between GBM and normal brain tissue. Interestingly, expression of ASL, GATM, ODC, SLC25A13 and SLC25A15 is increased in tumours compared to normal brain tissue (Figure 6a), indicating that the arginine biosynthesis pathway could be highly active in human brain tumours. However, this analysis also revealed that ASS1 and NOS are markedly downregulated in tumour tissue compared to normal brain (Figure 6a).

To validate the differential expression of the TCGA dataset genes in the cell culture system used here, we performed qPCR analyses in NS and ML from U87 and NCH644 cells. From the 7 genes included in this panel, *NOS1* and *GATM* were not detected by qPCR, most likely due to very low expression. Importantly, *ASS1* expression was significantly upregulated in U87 NS compared to ML (Figure 6b), while being significantly downregulated in NCH644 NS compared to ML (Figure 6c). *ASS1* downregulation was also detected in the U251 GBM cell line when exposed to NS culture (Figure S1a), thus recapitulating the results from NCH644 and human GBM tumours. Furthermore, NCH644 NS presented an upregulation of *ODC1*, *SLC25A15* and *SLC25A13* (Figure 6c), although the latter did not reach significance. Together, these results confirm the findings from our RNA sequencing analysis and suggest that human GBM tumours employ similar metabolic strategies to those identified by our study. Since NCH644 NS display reduced expression of ASS1 with high expression of *ODC*, *SLC25A13* and *SLC25A15*, it could be concluded that the specific metabolic routes used by these cells in NS culture seem to be more representative of human GBM tumours and thus may better resemble the in vivo setting of the disease. In contrast, U87 NS showed upregulation of *ASS1*, while *ASL*, *ODC1*, *SLC25A13* and *SLC25A15* were downregulated (Figure 6b), indicating that these cells employ different metabolic strategies to adapt to these culture conditions. Of note, we observed that in U87 cells, both NS number and size were significantly reduced by using the ASS1 inhibitor α-methyl-DL-aspartic acid (MDLA) [15,16,17,18] (Figure S1b). This effect could be rescued by the addition of arginine to the culture medium (Figure S1b), indicating that U87 NS indeed require de novo arginine biosynthesis by ASS1.

**Figure 7:**
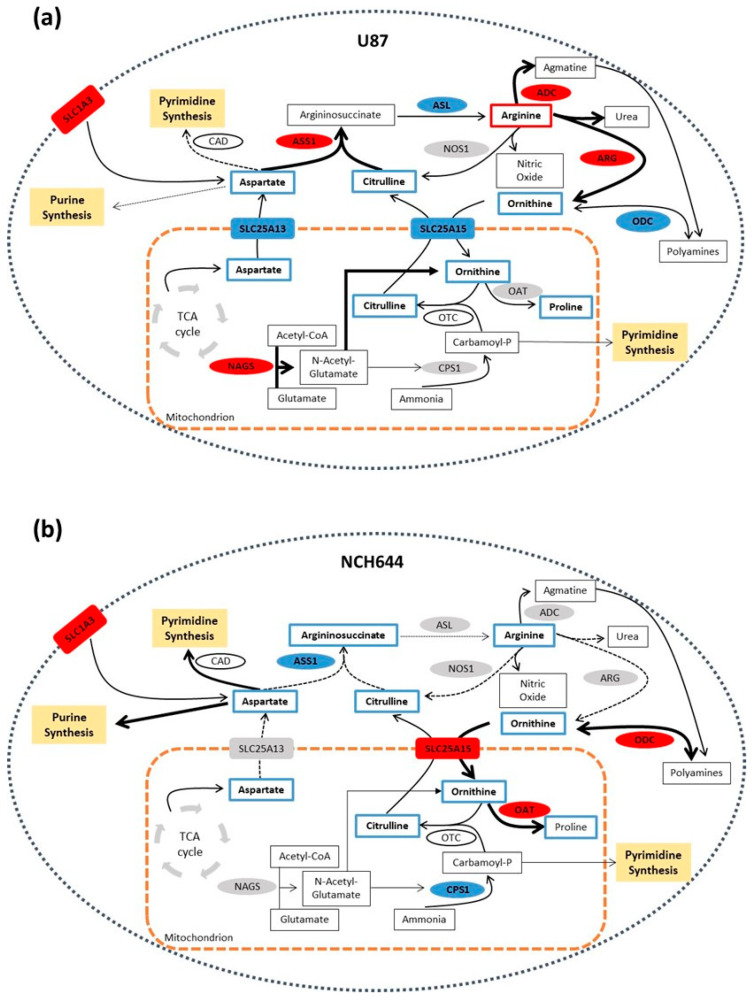
Proposed model of arginine metabolism in U87 and NCH644 neurospheres. (**a**) U87 NS present ASS1 upregulation, pointing towards aspartate shuttling into arginine synthesis. Arginine may then be used by ADC or ARG in the cytoplasm for agmatine and ornithine production, respectively. By upregulating NAGS, ornithine production may be further induced to maintain a high flux of arginine synthesis. Increased arginine synthesis and urea cycle metabolism in these cells may result in a reduction of nucleotide metabolism, by reducing the available aspartate pool and preventing purine and pyrimidine synthesis. (**b**) NCH644 NS, by downregulating ASS1, may shuttle aspartate away from arginine biosynthesis and towards nucleotide production. In addition, ornithine may be used by ODC in the cytoplasm for the production of polyamines or it can enter the mitochondria through the SLC25A15 transporter to be used by OAT for proline synthesis. Through ASS1-dependent metabolic rewiring, NCH644 cells could avoid feeding aspartate into the urea cycle and preserve the availability of this amino acid for purine and pyrimidine synthesis, thus supporting their growth. ADC—arginine decarboxylase; ARG—arginase; ASL—argininosuccinate lyase; ASS1—argininosuccinate synthase; CAD—carbamoyl-phosphate synthetase 2, aspartate transcarbamylase and dihydroorotase; CPS1—carbamoyl-phosphate synthase 1; NAGS—N-acetylglutamate synthase; NOS1—nitric oxide synthase 1; OAT—ornithine aminotransferase; ODC1—ornithine decarboxylase 1; SLC1A3—solute carrier family 1 member 3; SLC25A13—solute carrier family 25 member 13; SLC25A15—solute carrier family 25 member 15.

Taken together, these results suggest that although the same metabolic pathway is significantly regulated in NS from different GBM cell lines, the exact metabolic wiring of this pathway can be modulated in a highly specific manner to achieve different metabolic needs. U87 cells exposed to NS conditions show a clear downregulation of proliferation, thereby reducing their demand for nucleotide biosynthesis. They upregulate ASS1 to divert aspartate into the urea cycle for arginine biosynthesis (Figure 7a). In contrast, NCH644 cells exposed to NS conditions, where they continue to proliferate, downregulate ASS1 to prevent the funnelling of aspartate into the urea cycle. Instead, aspartate is shuttled away from arginine biosynthesis, potentially towards nucleotide biosynthesis to sustain proliferation (Figure 7b).

## 3. Discussion

It is widely recognised that defined culture conditions enabling the formation of NS lead to an enrichment of stem-like properties in GBM cells. These stem-like cells can be functionally distinguished from their differentiated counterparts at many levels, ranging from gene expression to metabolic regulation. The results of our study support this notion by demonstrating that exposing GBM cells to different culture conditions causes global rewiring of their transcriptome and metabolome, with a high degree of separation shown by PCA and hierarchical clustering. Although U87 cells are adapted to grow under ML conditions [19,20], it is evident that NS culture selects for a stem-like phenotype, evidenced by the induction of stem cell-related gene signatures and the downregulation of differentiation-related gene sets. Similarly, NCH644 cells, which are adapted to grow under NS conditions [20,21,22,23], also showed downregulation of the same differentiation-related gene sets, supporting the conclusion that the different culture conditions indeed select for a more stem-like or differentiated state. Thus, both U87 and NCH644 cells seem to be appropriate cellular models to study stem-like and differentiated states in GBM. Indeed, NS from both cell lines have been extensively studied as stem-like cell model systems, both in vitro and in vivo [19,20,21,22,23,24,25,26].

Since the main purpose of this work was to study metabolic similarities and differences between U87 and NCH644 cells under ML vs. NS conditions, our approach was to perform general profiling of the cells using both transcriptomics and LC-MS-based metabolomics, followed by detailed pathway analysis using the MetaboAnalyst platform [27]. Several studies have already characterised different aspects of the metabolic features of GSCs [13,21,28,29,30,31]. Zhou et al. found that cancer stem-like cells (CSCs) isolated from human GBM xenografts display increased glycolysis and low mitochondrial respiration through downregulation of succinate dehydrogenase subunit B (SDHB), leading to increased electron leakage and reactive oxygen species (ROS) production [13]. In other studies, enzymes involved in glycolysis, namely, pyruvate kinase M2 (PKM2) and 6-Phosphofructo-2-Kinase/Fructose-2,6-Biphosphatase 4 (PFKFB4), were identified as stemness regulators in GSCs [21,32]. Additionally, Mao et al. found that two distinct tumour-derived GSC subtypes—proneural and mesenchymal GSCs—were prominently associated with the clinically recognised proneural and mesenchymal subtypes of GBM and had distinct dysregulated signalling pathways [31]. Mesenchymal GSCs highly expressed enzymes of the aldehyde dehydrogenase (ALDH) family, especially ALDH1A3 that is involved in glycolysis. Inhibition of ALDH1A3 attenuated the growth of mesenchymal but not proneural GSCs, suggesting that different CSC populations may employ distinct stemness-regulating metabolic phenotypes [31]. Highlighting their metabolic heterogeneity, GSCs were also reported to consume less glucose and produce less lactate while having higher ATP levels, when compared with differentiated cancer cells [30]. Moreover, inhibition of either glycolysis or mitochondrial respiration in GSCs had minimal effect on energy production and only the combined inhibition of both pathways was able to deplete intracellular ATP levels. GSCs revealed features of metabolic plasticity, indicating that targeting individual metabolic pathways may not be sufficient to eradicate these cells [30]. Although some of these findings still require preclinical evaluation, it is clear that targeting the metabolic vulnerabilities of GSCs could hold promise for future cancer therapies.

In our study, the general metabolome analysis revealed that the vast majority of metabolites that were concomitantly regulated in both U87 and NCH644 cells were found to be downregulated in NS compared to ML cultures. These included TCA cycle metabolites (citrate, α-ketoglutarate, succinate, fumarate and malate), amino acids (alanine, aspartate, isoleucine, citrulline, glutamate, histidine, methionine, ornithine, phenylalanine, proline, sarcosine, serine, threonine and tyrosine) and purine/pyrimidine metabolism-related metabolites (xanthine, xanthosine, UDP, thymidine and thymine), suggesting that NS of both U87 and NCH644 cells may exhibit a reduced overall metabolic activity, compared to cells in ML culture. On the other hand, the only metabolite that presented higher levels in NS from both U87 and NCH644 was hypoxanthine, which points to a possible role of this metabolite in stem-like cells. Hypoxanthine is converted into inosine monophosphate (IMP) by the enzyme hypoxanthine guanine phosphoribosyltransferase (HPRT), which plays a major role in the salvage pathway for purine biosynthesis. Thus, hypoxanthine accumulation could be indicative of an impaired salvage pathway and an enhanced dependency on de novo purine synthesis in NS. Indeed, despite the existence of two alternative routes for purine biosynthesis, i.e., via de novo synthesis or via the salvage pathway, many cancer cells display a high dependence on the de novo nucleotide synthesis [33,34]. Interestingly, U87 NS showed significantly higher levels of guanine and guanosine, possibly due to an increase in the de novo purine synthesis. In contrast, NS from NCH644 displayed lower levels of guanine and guanosine, suggesting that these metabolites are either being produced at a low rate or rapidly metabolised in these cells. It is possible that the HPRT enzyme may be using guanine for the production of guanine monophosphate (GMP) in NCH644 cells instead of using hypoxanthine for IMP synthesis via the salvage pathway. Since the salvage pathway skips energetically expensive steps of de novo nucleotide biosynthesis [35], the production of purines via the salvage pathway in NCH644 NS may be more energy-efficient compared to U87 NS. This may also support the highly proliferative state of NCH644 cells in NS cultures and prevent the induction of stress and apoptosis. However, it has to be noted that our results only represent a snapshot of metabolite levels in the cells, and the observed decrease in guanine and guanosine levels in NCH644 NS could also be indicative of their increased demand for nucleotides for rapid proliferation. Although these hypotheses were not functionally tested in this study, they are in line with the observed changes in gene expression in U87 and NCH644 cells. While U87 cells show reduced proliferation in NS compared to ML cultures, this was not observed in NCH644 cells. In fact, U87 cells displayed several altered gene sets indicating reduced proliferation. These included gene sets related to proliferation (cell cycle, translation, DNA synthesis and repair) and metabolism (TCA cycle, mitochondrial activity, PPP and glutamine metabolism), which were all downregulated in U87 NS. Moreover, gene signatures associated with apoptosis and the negative regulation of nucleotide metabolism were upregulated in U87 NS, indicating an overall lower cellular activity. In contrast, NCH644 NS presented downregulation of apoptosis and oxidative stress gene sets, suggesting that NS cultures of this cell line have an increased vitality. In fact, it was described that basic fibroblast growth factor (bFGF) in the NS culture medium leads to resistance to apoptosis in NCH644 cells [23]. Thus, adaptation to specific culture conditions may explain why U87 cells, which are not adapted to grow in NS culture, and NCH644 cells, which are not adapted to grow under ML conditions, may display reduced proliferation when challenged with the other culture condition.

Despite these differences, the global metabolic adaptations triggered by NS conditions showed a surprising overlap between the two cell lines, as both types of analysis, either based on metabolites only or a joint analysis of metabolomic and transcriptome data, identified similar metabolic processes that are differentially regulated between ML and NS cultures in both U87 and NCH644 cells. In particular, “Arginine biosynthesis” was the most prominently regulated pathway in both cell lines. It is well established that tumour cells have high demands for nutrients to fuel rapid growth and proliferation and arginine is one of the conditionally essential amino acids that is required by many malignant cells. Increased arginine biosynthesis due to the upregulation of ASS1 has been reported in ovarian, colorectal, gastric and lung cancers, compared to the corresponding normal tissues [15,36,37,38]. While the role of ASS1 in cancer as well as the molecular mechanism mediating ASS1 upregulation remains unclear, it has been described that high *ASS1* expression is associated with increased proliferation and tumourigenicity of colorectal cancer [39]. In addition, inhibition of ASS1 expression in combination with arginine depletion in gastric cancer has been shown to inhibit cancer cell migration and motility, therefore affecting metastasis formation in vivo [15].

However, a number of human cancers, including melanoma, hepatocellular carcinoma and prostate carcinoma, are defined as auxotroph for arginine, indicating that these tumours are unable to synthesise arginine de novo, due to ASS1 deficiency [40]. Consequently, arginine deprivation has been shown to be effective as an adjuvant treatment for tumours that lack de novo arginine synthesis [41]. The effectiveness of arginine catabolising agents has been reported in several cancer models, including glioblastoma [42], breast [43], osteosarcoma [44], bladder [45], ovarian [46] or pancreatic cancers [47]. On the other hand, arginine is also a critical metabolite for immune cell function and required for the efficient elimination of cancer cells [48]. Therefore, arginine supplementation was associated with tumour growth inhibition by enhancing innate and adaptive immune responses [49,50,51].

It has been reported that downregulation of urea cycle metabolism, specifically due to decreased expression and activity of ASS1, diverts aspartate towards pyrimidine biosynthesis and supports cell proliferation as part of the metabolic reprogramming in cancer [52,53]. Furthermore, inhibition of *ASS1* via epigenetic regulation was also reported in GBM, specifically in CD133-positive GSCs [42]. In our study, we found that NS of NCH644 cells show a dramatic downregulation of *ASS1* compared to ML cultures, and this regulation was also confirmed in U251 cells. Moreover, TCGA data analysis also revealed a prominent *ASS1* downregulation in tumour samples from human GBM patients compared to normal brain tissue. This suggests that NCH644 cells in NS cultures could employ similar metabolic routes to those found in the human disease. In fact, qPCR analysis of several genes coding for enzymes linked to arginine metabolism showed broadly overlapping results between NCH644 NS cultures and GBM tumours from the TCGA dataset. While *ASS1* was found to be downregulated in GBM tumours and NCH644 NS cultures, the expression of *ODC1*, *SLC25A13* and *SLC25A15* was increased. In contrast, U87 NS displayed upregulation of *ASS1* and concomitant downregulation of *ASL*, *ODC1*, *SLC25A13* and *SLC25A15*. Thus, it can be concluded that NS cultures of NCH644 cells recapitulate some of the metabolic features also found in human tumours and may therefore represent a suitable model to study metabolic reprogramming in GSCs. The results of our work suggest that arginine biosynthesis may be an important metabolic pathway in the stimulation and maintenance of the stem-like phenotype. Nevertheless, there were clear differences in the regulation of specific components of this pathway between the two cell lines. Indeed, the significant upregulation of *ASS1* in U87 NS and the downregulation of the same gene in NCH644 NS was the most prominent difference in the regulation of this pathway. Interestingly, the SLC25A15 transporter, which transports ornithine from the cytoplasm to the mitochondria, was found to be significantly upregulated in NCH644 NS, suggesting that the low amount of ornithine in NCH644 NS may be directed to the mitochondria and thus away from arginine biosynthesis. Although U87 NS also display significantly reduced levels of ornithine, this reduction was not as strong as in NCH644 NS, possibly due to the high expression of ARG2 and NAGS, which maintain an influx of ornithine into the urea cycle for arginine biosynthesis.

In addition to defining arginine provision, differences in *ASS1* regulation in the two cellular systems also determine the metabolic fate of aspartate. Aspartate can be used for the synthesis of arginine as part of the cytoplasmic arm of the urea cycle. Through the ATP-dependent activity of ASS1, aspartate is condensed with citrulline to form argininosuccinate and subsequently cleaved into arginine and fumarate by argininosuccinate lyase (ASL). Aspartate can either be synthesised from the TCA cycle metabolite oxaloacetate and then transported to the cytoplasm by the mitochondrial SLC25A13 transporter or taken up by the cells through the SLC1A3 transporter. Since aspartate is an essential precursor for nucleotide synthesis, the conversion of aspartate to argininosuccinate by ASS1 can deplete the intracellular pool of this metabolite, thereby limiting its availability for nucleotide synthesis and resulting in reduced proliferation. Despite the differences in the regulation of *ASS1*, we found that both U87 and NCH644 NS displayed significantly lower levels of aspartate compared to ML cultures. Additionally, expression of the high-affinity amino acid transporter SLC1A3, that mediates the uptake of glutamate and aspartate by cells, was significantly increased in NS of both U87 and NCH644 cells, albeit much stronger in U87. This may suggest that not only aspartate provision but also its metabolic fate may be very different in the two cell lines. In U87 NS, aspartate may be preferentially shuttled towards arginine biosynthesis, while NS of NCH644 cells could use aspartate mainly for nucleotide synthesis. In line with our finding, it can be speculated that arginine deprivation would impair growth in NCH644 NS and U87 ML cultures, due to ASS1 downregulation and the resulting auxotrophy for arginine. On the other hand, U87 NS and NCH644 ML cultures would not benefit from arginine deprivation, since they display ASS1 upregulation and thus may be able to synthesise arginine de novo. ASS1 regulation may thus define the susceptibility of stem-like or differentiated cancer cells towards therapies targeting arginine biosynthesis. Indeed, we have already shown that the growth of U87 NS was significantly impaired when cells were treated with the ASS1 inhibitor MDLA.

Our results identify arginine metabolism as a key metabolic process associated with NS culture in two different GBM cellular systems. While altered arginine metabolism is associated with conditions that enrich for stem-like cells, the exact metabolic rewiring of this metabolic process is highly specific. The distinct adaptations found in each cell line are in line with their specific metabolic demands. Thus, the use of different metabolic routes within this pathway ensures cell proliferation and survival by fulfilling the metabolic needs of each cell system through transcriptional and metabolic adaptations. Moreover, the overlap in the observed changes in expression of arginine biosynthesis genes between NCH644 NS and data from human tumours indicate similar rewiring of this pathway. Our results indicate that GSCs may exhibit classical auxotrophy for arginine and suggest that the use of arginine-depriving therapies could be beneficial for GBM patients by potentially targeting and eliminating GSCs.

## 4. Materials and Methods

### 4.1. Glioblastoma Cell Lines

The cell lines U87 and U251 were obtained from the American Type Culture Collection (ATCC^®^, Manassas, VA, USA) and the glioblastoma stem-like cell line NCH644 was purchased from Cell Line Services (CLS, Eppelheim, Germany).

### 4.2. Cell Culture in Monolayer Conditions

U87 and U251 cells were grown in 10 cm dishes with DMEM high glucose (Sigma-Aldrich 6546, St. Louis, MO, USA) supplemented with 10% FBS (Sigma-Aldrich 7524, St. Louis, MO, USA), 2 mM glutamine (Sigma-Aldrich 59202C, St. Louis, MO, USA) and 1% penicillin/streptomycin (Sigma-Aldrich 4333, St. Louis, MO, USA). NCH644 cells, normally grown under neurosphere (NS) conditions, were transferred to monolayer (ML) conditions by singularising NS with accutase and plating them in 10 cm dishes with DMEM high glucose (Sigma-Aldrich 6546, St. Louis, MO, USA) supplemented with 10% FBS (Sigma-Aldrich 7524, St. Louis, MO, USA), 2 mM glutamine (Sigma-Aldrich 59202C, St. Louis, MO, USA) and 1% penicillin/streptomycin (Sigma-Aldrich 4333, St. Louis, MO, USA). Cells were maintained in a cell incubator at 37 °C, 5% CO_2_ and a relative humidity of 95%. Specific culture conditions used for transcriptomics and metabolomics are described in Section 4.4 and Section 4.6, respectively.

### 4.3. Cell Culture for Neurosphere Formation

For NS formation of U87 and U251 cell lines, cells were plated in low adhesion 6-well plates coated with poly-HEMA (Poly 2-hydroxyethyl methacrylate; Sigma-Aldrich P3932, St. Louis, MO, USA), with DMEM F12 (Life technologies 21331046, Carlsbad, CA, USA) supplemented with 2 mM glutamine (Sigma-Aldrich 59202C, St. Louis, MO, USA), 1% penicillin/streptomycin (Sigma-Aldrich 4333, St. Louis, MO, USA), 20 ng/mL bFGF (Peprotech 100-18B, London, UK), 20 ng/mL EGF (Peprotech AF-100-15, London, UK), 10 ng/mL LIF (Peprotech 0300-05, London, UK) and 1x B27 (Thermo Fisher Scientific 12587010, Waltham, MA, USA), and maintained in a cell incubator at 37 °C, 5% CO_2_ and a relative humidity of 95%. 

For NS formation of NCH644 cell line, cells were plated in hydrophobic surface T75 flasks (Sarstedt, Nümbrecht, Germany) with DMEM F12 (Life technologies 21331046, Carlsbad, CA, USA) supplemented with 2 mM glutamine (Sigma-Aldrich 59202C, St. Louis, MO, USA), 1% penicillin/streptomycin (Sigma-Aldrich 4333, St. Louis, MO, USA), 20 ng/mL bFGF (Biomol 50,361.50, Hamburg, Germany), 20 ng/mL EGF (Invitrogen PHG0311, Carlsbad, CA, USA) and 2% BIT Admixture (Pelo Biotech PB-SH-033-0000, Planegg/Martinsried, Germany) and maintained in a cell incubator at 37°C, 5% CO_2_ and a relative humidity of 95%. Specific culture conditions used for transcriptomics and metabolomics are described in Section 4.4 and Section 4.6, respectively.

For α-methyl-DL-aspartic acid (MDLA; Sigma-Aldrich M6001, St. Louis, MO, USA) and arginine (Sigma-Aldrich A5006, St. Louis, MO, USA) treatments, neurosphere formation was performed in 24-well plates coated with poly-HEMA, by plating 5000 cells with 0.5 mL medium per well. Additionally, 0.5% methylcellulose (Sigma-Aldrich M0512, St. Louis, MO, USA) DMEM F12 medium was used, supplemented with all the components mentioned above. Cells were treated with 5 mM MDLA and/or 3 mM arginine and maintained in a cell incubator for about 8 to 10 days. The number of neurospheres formed per well was counted and the neurospheres’ size was quantified by ImageJ software.

### 4.4. RNA Sequencing

The experiment was performed in triplicate for each condition. For NS assay, 20,000 U87 cells were cultured with 2 mL serum-free medium per well in two 6-well plates and 500,000 NCH644 cells in a T75 flask with 10 mL serum-free medium. After 4 days, NS cells were collected, centrifuged at 700 rpm for 3 min and the medium was removed. For ML cultures, 10 cm dishes were used to culture 500,000 U87 and NCH644 cells with 10 mL medium. After 4 days, the medium was removed and the cells were scraped. RNA was isolated with the RNeasy Mini Kit with additional DNase I digestion according to manufacturer’s protocol (Qiagen, Hilden, Germany). RNA was eluted in 40 μL RNAase free H_2_O and kept on ice and concentration was measured using a standard sense chip for Bioanalyzer (Agilent, Santa Clara, CA, USA). One μg RNA per each condition was used for library preparation. The library was prepared using the NEBNext Ultra RNA library prep kit for Illumina (San Diego, CA, USA) and the NEBNext Multiplex Oligos for Illumina (Dual Index Primers Set 1), according to the manufacturer’s instructions. H_2_O was used as negative control to check the purity of the preparation. Amplification by PCR was performed for 12 cycles and the PCR products were purified using the Agencourt AMPure XP beads. The quality of the purified DNA was verified on the Bioanalyzer (Agilent, Santa Clara, CA, USA) before performing the sequencing, using the Illumina GAIIx sequencer (San Diego, CA, USA). All samples were mixed at equimolar concentration of 300 nM.

Base calling was performed with CASAVA software (1.7, Illumina, San Diego, CA, USA) and overall sequencing quality was tested using the FastQC script. Reads were aligned to the human genome (hg19) with TopHat2 and Bowtie v0.12.8 using default parameters [54,55]. Mapped reads per gene (EnsEMBL GRCh37, release 74) were counted using the “summarizeOverlaps” function in the GenomicAlignments R package, non-expressed genes were removed (mean read count per gene over all samples > 1) and TMM-normalised with EdgeR. Gene set enrichment analysis was performed with the C2, C5 and Hallmark collection from the MSigDB v5.2 (UC San Diego, San Diego, CA and Broad Institute, Cambridge, MA, USA) with default parameters and 1000 permutations [56,57]. 

### 4.5. qPCR Analysis

RNA was isolated using the RNeasy Mini Kit with additional DNase I digestion according to manufacturer’s protocol (Qiagen, Hilden, Germany), followed by reverse transcription into complementary DNA (cDNA) using RevertAid First Strand cDNA Synthesis Kit (Thermo Fisher Scientific, Waltham, MA, USA). Real-time PCR was performed using Power SYBR™ Green PCR Master Mix (Thermo Fisher Scientific, Waltham, MA, USA) and custom primers (Integrated DNA Technologies, Coralville, IA, USA). Relative mRNA levels were calculated after normalisation to B2M, using the double Delta Ct method. qPCR reactions were performed in technical triplicate on three biologically independent samples. Primers sequences are the following: human ASS1 forward 5′-CCAGCAGGCACCATCCTTTA-3′ and reverse 5′-CGCACTTCCCGGTCCAT-3′; human ASL forward 5′-GACACTATCCGTGCGGCCA-3′ and reverse 5′-AAAGCTTCCCACTCTCCGAGG-3′; human ODC1 forward 5′-CCTGGGCGCTCTGAGATTGT-3′ and reverse 5′-CAGCTTCTCACAAAGGCAACTC-3′; human SLC25A13 forward 5′-GACTAGAAGTGAGCCGCCC-3′ and reverse 5′-TGGTTAAAGCCACCTTGGCG-3′; human SLC25A15 forward 5′-AGCCAAGAGCCAGAATACAG-3′ and reverse 5′-AGAGTCCATGGTAGAACCCCA-3′; human NOS1 forward 5′-TTTATGCCGCGTTTCCAGCC-3′ and reverse 5′-GCATCATGAGCCCGTCCG-3′; human GATM forward 5′-AGAGGCCAGGAACATTCCGC-3′ and reverse 5′-AGGTTCGTCCAAGCCGAGAT-3′; human B2M forward 5′- GTGCTCGCGCTACTCTCTC-3′ and reverse 5′- GTCAACTTCAATGTCGGAT-3′.

### 4.6. Extraction of Water-Soluble Metabolites from Cells

For metabolite extraction of NS, 20,000 U87 cells were plated per well of a 6-well plate and 500,000 NCH644 cells were plated in a T75 flask. After 4 days, 0.5 mL ice-cold methanol/water (80/20, *v*/*v*) containing 0.25 μM of the internal standard lamivudine (Sigma-Aldrich, St. Louis, MO, USA) was added to collected, washed and snap-frozen NS. For metabolite extraction of ML cells, 500,000 U87 and NCH644 cells were plated in a 10 cm dish. After 4 days, cells were washed with cold 154 mM ammonium acetate, snap frozen in liquid nitrogen and scraped off after addition of 0.5 mL ice-cold methanol/water (80/20, *v*/*v*) containing 0.25 μM of the internal standard lamivudine (Sigma-Aldrich, St. Louis, MO, USA). The resulting suspension was transferred to a reaction tube, mixed vigorously and centrifuged (2 min, 16,000× *g*). Supernatants were transferred to a Strata^®^ C18-E column (Phenomenex, Torrance, CA, USA) which were previously activated with 1 mL of CH_3_CN and equilibrated with 1 mL of MeOH/H_2_O (80/20, *v*/*v*). The eluate was evaporated in a vacuum concentrator. The resulting residue was dissolved in 50 μL 5 mM NH_4_OAc in CH_3_CN/H_2_O (25/75, *v*/*v*). The pellets were kept for protein determination.

### 4.7. Liquid Chromatography–Mass Spectrometry (LC–MS)

Samples were diluted 1:2 with CH_3_CN and 5 μL of each sample was applied to a HILIC column (Acclaim Mixed-Mode HILIC-1, 3 μm, 2.1 × 150 mm). Metabolites were separated at 30 °C by LC using a DIONEX Ultimate 3000 UPLC system and the following solvents: Solvent A consisting of 5 mM NH_4_OAc in CH_3_CN/H_2_O (5/95, *v*/*v*) and solvent B consisting of 5 mM NH_4_OAc in CH_3_CN/H_2_O (95/5, *v*/*v*). The LC gradient program was: 100% solvent B for 1 min, followed by a linear decrease to 40% solvent B within 5 min, then maintaining 40% B for 13 min, then returning to 100% B in 1 min and 5 min 100% solvent B for column equilibration before each injection. The flow rate was maintained at 350 μL/min. The eluent was directed to the hESI source of the Q Exactive mass spectrometer (QE-MS) from 1.85 min to 18.0 min after sample injection (Thermo Fisher Scientific Waltham, MA, USA). The scan range was set to 69.0 to 1000 *m*/*z* with a resolution of 70,000 and polarity switching (negative and positive ionisation). Peaks corresponding to the calculated metabolites masses taken from an in-house metabolite library (MIM +/− H^+^ ± 2 mmU) were integrated using TraceFinder software (Thermo Fisher Scientific, Waltham, MA, USA).

### 4.8. Metabolomics and Transcriptomics Analyses

Statistical analyses were performed using R (version 4.0.2, Free Software Foundation’s GNU project, Boston, MA, USA) and MetaboAnalyst 4.0 [27]. Separate statistical analyses of transcriptomics and metabolomics data were performed using the “General Statistical Analysis and Visualization” option in MetaboAnalyst. PCA 2D scoreplots, heatmaps (Distance measure: Euclidean, Algorithm: Ward) and dendrograms (Distance measure: Euclidean, Algorithm: Ward) were exported in svg format. For joint pathway analysis, significantly changed metabolites and transcripts were selected (*p* < 0.05, FDR-corrected two-sided Student’s *t*-test). Metabolite names were translated into KEGG IDs prior to analysis. Log2FCs of transcripts and metabolites were submitted for analysis and the correct organism (homo sapiens) was selected (Algorithm specifications: Enrichment analysis—“Hypergeometric test”, Topology measure—“Degree Centrality”, Integration method—“Combine query”). Result tables and pathway overview plots were exported for “Metabolic Pathways—Metabolites Only” and “Metabolic Pathways—Integrated”. Volcano plots were generated using the ggplot2 package in R (version 4.0.2, Free Software Foundation’s GNU project, Boston, MA, USA).

### 4.9. Analysis of TCGA Data

RNA-seq datasets from human glioblastoma tumours from The Cancer Genome Atlas were accessed through the GEPIA tool (http://gepia.cancer-pku.cn, accessed on 3 December 2020). Data from normal tissue are provided from the Genotype-Tissue Expression (GTEx) project (https://gtexportal.org, accessed on 3 December 2020). Data were selected based on a log2-fold change cut-off of 1 and a *p*-value cut-off of 0.01 [14].

## 5. Conclusions

Using two different culture conditions in two GBM cell lines, U87 and NCH644, we have shown here that the induction of a stem-like phenotype induces the remodelling of specific metabolic pathways, and that the metabolic portrait of GSCs is fundamentally different from that of cells in a more differentiated state. Importantly, the two cell lines used in our study adapted to the different culture conditions—monolayer cultures that induce a more differentiated state and neurosphere cultures that select for stem-like cells -, by displaying overlapping metabolic alterations, in particular, deregulation of the arginine biosynthesis pathway. However, the regulation of individual nodes within this pathway in the two cell lines resulted in different metabolic outcomes, potentially linked to the specific metabolic demands of each cell line. Overall, U87 cells responded to growth conditions favouring a stem-like state by upregulating the enzyme ASS1 accompanied by significantly elevated levels of arginine along with the downregulation of gene signatures associated with cell cycle progression, DNA synthesis and PPP activity. This supports the notion that these cells have a reduced requirement of aspartate as a precursor for nucleotide biosynthesis and instead shuttle this metabolite towards arginine generation and the urea cycle. In line with this conclusion, we observed that inhibition of ASS1 using a pharmacological inhibitor reduced NS formation in U87 cells, thus showing the high dependence of these cells on arginine synthesis. In contrast, NCH644 cultures enriched for stem-like cells showed a substantial downregulation of ASS1 and had significantly lower levels of arginine. Moreover, these cells did not downregulate gene signatures associated with DNA synthesis, cell cycle and proliferation when compared to their more differentiated counterparts, indicating that this cell line maintains higher proliferative potential under stem-like-cell-enriched culture conditions. This suggests that these cells direct a higher proportion of aspartate towards nucleotide biosynthesis and have a lower dependence on arginine synthesis. However, further experiments, for example, using stable isotope tracing techniques, are required to determine the exact fate of specific metabolites in the two cellular systems. Moreover, further exploration could determine how arginine metabolism is connected to the stem-like state and disclose the complete metabolic rewiring in GBM stem-like cells. Importantly, several enzymes involved in arginine biosynthesis, including ASS1, were also found to be significantly regulated in human GBM, suggesting that NCH644 cells may more closely resemble the in vivo setting of this disease and should be used for further studies addressing the therapeutic benefits of targeting this metabolic pathway.

## Data Availability

The data presented in this study are available in this article (and Supplementary Material).

## Supplementary Materials

The following are available online at https://www.mdpi.com/2072-6694/13/6/1327/s1, Figure S1: ASS1 expression in ML and NS cultures of U251 cells and MDLA effect on U87 NS cultures; Table S1: Transcriptomics and metabolomics data.
