# Supplementary material for: Integrated Metabolomics and Transcriptomics Analysis of Monolayer and Neurospheres from Established Glioblastoma Cell Lines
Source: Cancers (Basel). 2021 Mar 16;13(6):1327. doi: 10.3390/cancers13061327 (PMC8001840; doi:10.3390/cancers13061327)
Supplement: Supplementary file 1 [file cancers-13-01327-s001.zip › supplementary/cancers-1142787-crosschek-SUPPLE-final.docx]

Supplementary Material: Integrated Metabolomics and
Transcriptomics Analysis of Monolayer and Neurospheres from Established Glioblastoma Cell Lines

Joana Peixoto, Sudha Janaki-Raman, Lisa Schlicker, Werner Schmitz, Susanne Walz, Alina M. Winkelkotte, Christel Herold-Mende, Paula Soares, Almut Schulze and Jorge Lima


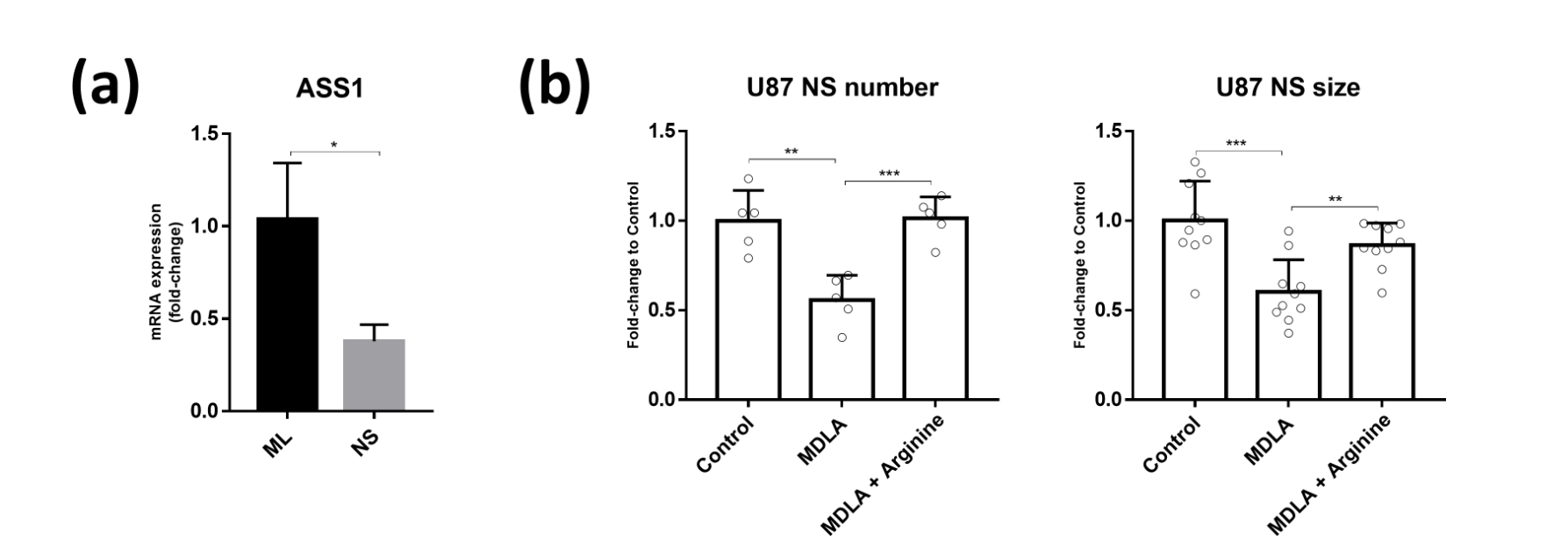


**Figure S1.** ASS1 expression in ML and NS cultures of U251 cells and MDLA effect on U87 NS cultures. (**a**) ASS1 mRNA expression in ML and NS cultures of U251 cells. (**b**) MDLA and arginine treatment effect on the number and size of U87 NS culture. * *p*<0.05, ** *p*<0.01, *** *p*<0.001
